# Transcriptome Reveals Allele Contribution to Heterosis in Maize

**DOI:** 10.3389/fpls.2021.739072

**Published:** 2021-09-23

**Authors:** Jianzhong Wu, Dequan Sun, Qian Zhao, Hongjun Yong, Degui Zhang, Zhuanfang Hao, Zhiqiang Zhou, Jienan Han, Xiaocong Zhang, Zhennan Xu, Xinhai Li, Mingshun Li, Jianfeng Weng

**Affiliations:** ^1^Institute of Crop Science, Chinese Academy of Agricultural Sciences, Beijing, China; ^2^Institute of Forage and Grassland Sciences, Heilongjiang Academy of Agricultural Sciences, Harbin, China; ^3^Institute of Crop Cultivation and Tillage, Heilongjiang Academy of Agricultural Sciences, Harbin, China

**Keywords:** heterosis, transcriptome, maize, allele, DEGs

## Abstract

Heterosis, which has greatly increased maize yields, is associated with gene expression patterns during key developmental stages that enhance hybrid phenotypes relative to parental phenotypes. Before heterosis can be more effectively used for crop improvement, hybrid maize developmental gene expression patterns must be better understood. Here, six maize hybrids, including the popular hybrid Zhengdan958 (ZC) from China, were studied. Maize hybrids created in-house were generated using an incomplete diallel cross (NCII)-based strategy from four elite inbred parental lines. Differential gene expression (DEG) profiles corresponding to three developmental stages revealed that hybrid partial expression patterns exhibited complementarity of expression of certain parental genes, with parental allelic expression patterns varying both qualitatively and quantitatively in hybrids. Single-parent expression (SPE) and parent-specific expression (PSE) types of qualitative variation were most prevalent, 43.73 and 41.07% of variation, respectively. Meanwhile, negative super-dominance (NSD) and positive super-dominance (PSD) types of quantitative variation were most prevalent, 31.06 and 24.30% of variation, respectively. During the early reproductive growth stage, the gene expression pattern differed markedly from other developmental stage patterns, with allelic expression patterns during seed development skewed toward low-value parental alleles in hybrid seeds exhibiting significant quantitative variation-associated superiority. Comparisons of qualitative gene expression variation rates between ZC and other hybrids revealed proportions of SPE-DEGs (41.36%) in ZC seed DEGs that significantly exceeded the average proportion of SPE-DEGs found in seeds of other hybrids (28.36%). Importantly, quantitative gene expression variation rate comparisons between ZC and hybrids, except for transgressive expression, revealed that the ZC rate exceeded the average rate for other hybrids, highlighting the importance of partial gene expression in heterosis. Moreover, enriched ZC DEGs exhibiting distinct tissue-specific expression patterns belonged to four biological pathways, including photosynthesis, plant hormone signal transduction, biology metabolism and biosynthesis. These results provide valuable technical insights for creating hybrids exhibiting strong heterosis.

## Introduction

Maize is an important crop that contributes significantly to the production of food and industrial raw materials around the world. Genetic improvement of maize mainly depended on its abundant genetic diversity and strong heterosis. Heterosis is a ubiquitous biological phenomenon in which hybrids exhibit superior performance relative to the biparental value of their parents for one or more traits. Researchers have been studying heterosis in depth from diverse perspectives. Environmental influences on heterosis (Duvick, 2005), which emphasize the environmental adaptability of plants to overcome the bottleneck of stresses. Combining ability effect tends to suggest that parental combining ability selection is the effective approach in hybrid breeding (Makumbi et al., 2011; Zhang et al., 2015). Genetic distance dominance (Wu et al., 2016) of heterosis suggests that the combination of combing ability tests and genetic relationship analysis can be used to define heterotic groups for breeding selection and genetic improvement. Changes in chromosome dose in multiple regions of the genome regulate quantitative traits, which reflect the influence of dose variation of chromosome dosage effects (Birchler et al., 2016). QTL interactions was used to analyze component accumulation of related quantitative traits could reasonably explain the genetic basis of part of heterosis (Xiang et al., 2016; Zhu et al., 2016). Allelic variation in gene expression might play a protective role in defense against adverse environments (Hoecker et al., 2008; Waters et al., 2017). “omics effects” can also be used to interpret heterosis at different levels (Huang et al., 2016; Alonso-Peral et al., 2017). Low DNA methylation was found in maize hybrids (where methylation inhibits gene expression), but not in rice as a whole, revealing the DNA methylation and epigenetic effects (Groszmann et al., 2013; Dapp et al., 2015). All of the hypotheses above attempt to reveal the genetic mechanisms controlling heterosis.

Although the exact molecular mechanisms behind heterosis remain unknown (Schnable and Springer, 2013), three competing, but not mutually exclusive, hypotheses propose that dominance effects, over-dominance effects, epistatic effects, or some combination thereof explain the genetic control of heterosis. Dominant hypothesis emphasizes the introduction of new ideal type dominant allele in the process of plant growth and development, the dominant allele more popular than the recessive allele (Davenport, 1908; Bruce, 1910; Jones, 1917). Over-dominance hypothesis emphasizes the complementarity between alleles of the offspring of hybrids, and holds that the heterozygous state is more favorable than the homozygous state (East, 1908; Shull, 1908; Larièpe et al., 2012). Epistatic interactions hypothesis emphasizes the favorable alleles in the role of heterosis gain. For any site, the effect can be produced by additive dominant or over-dominance (Minvielle, 1987; Schnell and Cockerham, 1992; Frascaroli et al., 2007). It should be noted that heterosis in self-pollinated species (e.g., rice) may involve different genetic interactions from heterosis in cross-pollinated species (e.g., maize), so both dominant and epistatic hypotheses may be related (Garcia et al., 2008).

Plant breeding is the aggregation of superior alleles from different germplasm sources. The aggregation of favorable alleles from different parents in the hybrids can exhibit much greater effects and may result in elite varieties (Cai et al., 2014). At present, intervarietal hybrid advantage is the main technical term used to describe unknown underlying mechanism(s) responsible for superior hybrid maize characteristics, with few mechanistic clues obtained from past studies that focused on maize germplasm-associated heterosis groups and patterns (Reif et al., 2003; Aguiar et al., 2008; Zhang et al., 2018; Annor et al., 2020). Nevertheless, early maize varieties cultivated in China possessed limited genetic diversity that has hindered attempts to substantially increase hybrid maize yields in recent decades. A large number of alleles were deleted by long-term artificial selection, caused losses in diversity as reflected by reduced allele numbers through elimination of unfavorable alleles (Gao et al., 2017). Indeed, the lack of adequate parental genetic variation has caused breeding efforts utilizing numerous heterosis groups to fail to improve grain yield, prompting researchers to investigate heterosis mechanisms associated with strong predominant hybrid maize.

The superior allele is the basis of the dominant expression in hybrids. Allelic specific expression in hybrids is one of the mechanisms of heterosis (Ma et al., 2021). Researchers have speculated that heterosis-associated gene expression may be influenced by multiple biological processes, including DNA sequence variation, gene copy number change, histone modification, transcription factor regulation and DNA methylation (Zarayeneh et al., 2017). In one study, large numbers of DEGs detected during differentiation of maize spikelets and florets were identified based on gene expression profile differences between hybrid Zhengdan 958 and its parental lines Zheng58 and Chang7-2. Intriguingly, this set of DEGs encoded transcription factors or enzymes involved in biosynthesis of stress-related metabolites, suggesting that such genes are key players in heterosis expression (Li et al., 2012). Meanwhile, altered parental gene expression patterns observed in hybrid offspring indicate that intergenerational differential gene expression can generate hybrid phenotypes that are either superior or inferior to parental phenotypes (Stupar et al., 2008). Most DEGs showed inconsistent expression in tissues, which suggested extensive variation in the regulation of gene expression in a tissue-specific manner, about 97% of the single-parent expressed (SPE) genes exhibited intermediate or transgressive expression in hybrids, which might provide a wide range of opportunities for hybrid complementation through heterosis (Zhou et al., 2019). From the perspective of genome composition, genes of hybrid originate from these of parental inbred lines, thus, changes in gene expression and regulation in hybrid offspring must be responsible for heterosis, as demonstrated in previous studies that compared gene expression profiles between hybrids and parent lines (Guo et al., 2004; Harrison et al., 2012; Paschold et al., 2012, 2014; Baldauf et al., 2016). However, studies comparing gene expression patterns among hybrids have not been reported.

The specific expression pattern of superior alleles is the molecular basis of heterosis to achieve. Hybrids can selectively express beneficial alleles under specific spatiotemporal conditions, thus showing superiority (Shao et al., 2019). Recently, transcriptomic analysis has served as a valuable tool for revealing regulatory mechanisms underlying differential expression of parental alleles and DEGs in hybrids vs. parental lines (Fu et al., 2015) and effects of parental genetic variations on expression of genes in hybrids (Hu et al., 2016; Li et al., 2016). Differential gene expression in hybrids may be responsible for heterosis, all possible patterns of gene action supports the hypothesis that multiple molecular mechanisms contribute to heterosis (Swanson-Wagner et al., 2006). The expression level of parental alleles in rice hybrids is unbalanced, which is considered to be one of the important mechanisms leading to heterosis (Shao et al., 2019; Lv et al., 2020). Therefore, the study of gene expression patterns in maize hybrids will play an important role in elucidating the heterosis mechanism. Although many hypotheses have been proposed regarding gene expression between parents and progeny that may reasonably explain one aspect of heterosis, but few studies investigating heterosis intensity of hybrids have been reported.

Regulation of gene expression runs through the whole development stage, but the significance of gene expression varies in stages of crop development. Many genes with specific functions are expressed only at a certain stage of plant growth and development, and their abnormal expression may lead to the generation of new phenotypes (Ferrándiz et al., 2000). Therefore, the study of gene expression changes in time and space is an important way to understand the mechanism of plant growth and development. Leaf, the most important site of photosynthesis in plants, is an essential source-to-sink sugar transport organ needed to sustain the entire reproductive process including grain yield. Indeed, leaf enlargement is a highly important characteristic of maize hybrids (Swanson-Wagner et al., 2006; Frascaroli et al., 2007). Maize spike differentiation, during which the plant meristem gradually shifts from vegetative to reproductive growth, is another key stage affecting maize yield. During maize spike differentiation, paired axillary spikelet meristem and floret meristem, which determine the number of rows and grains per ear, respectively, form and ultimately affect maize grain yield (Bommert et al., 2005; Pautler et al., 2013). Thus, leaves during the five-leaf stage, ears during the spikelet differentiation stage and seeds at 15 days post-pollination represent important transitions from maize vegetative to reproductive growth and are thus of great significance for breeding of high-yield maize.

Zhengdan958, a commercially successful hybrid variety created by Chinese maize breeding programs from parental inbred lines Zheng58 and Chang7-2 was studied here due to its expression of strong heterosis with regard to grain yield (Li et al., 2012; Kogelman et al., 2014; Yang et al., 2020). Over the past 10 years, this hybrid has been planted across China and is prized for its high and stable yield, high quality, resistance to high planting density, broad adaptability and resistance to multiple diseases and pests (Ma et al., 2018; Zhang et al., 2018). In this study, four typical maize inbred parental lines and six hybrids created from them using an incomplete diallel cross (NCII) strategy followed by comprehensive mRNA sequencing were used to investigate the effect of gene expression abundance of parental lines on hybrids, the variation of gene expression pattern among hybrids, and the specific expression gene and their pattern of Zhengdan958. Ultimately, results of this work should enhance our understanding of heterosis mechanisms so that heterosis can be harnessed to improve maize and other crops.

## Results

### Identification of Gene Expression Abundance in Four Parental Lines

**FIGURE 1:**
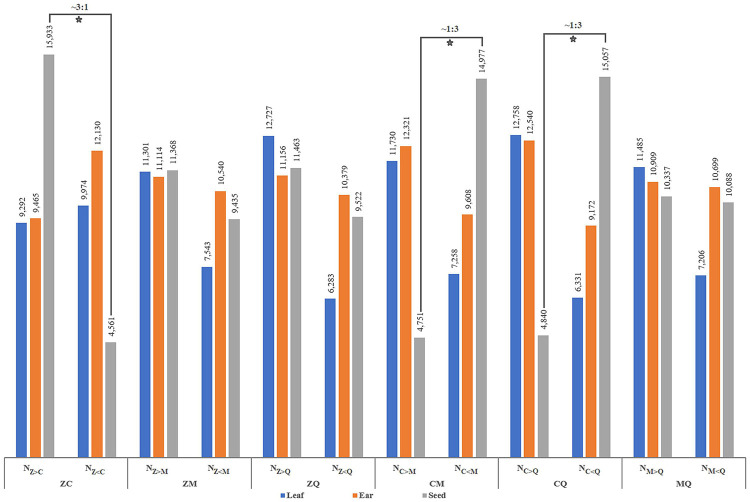
Variation in the number of DEGs between parental inbred lines. Nz_>_c denotes the number of genes whose value of FPKM is greater in parent Zheng58 than in parent Chang7-2, and the same goes for other acronyms. The numbers at the top of the bar chart represent the number of different types of DEGs, the hollow five-pointed stars show special proportions of the number of genes, and the tissues [leaves (L), ears (E), and seeds (S)] shown in different colors are listed on bottom.

Numbers of differentially expressed genes (DEGs) were similar between hybrids and inbred lines, with ear (E) the source of the highest number of DEGs and leaf (L) and seed (S) yielding basically similar numbers of DEGs (Supplementary Figure 1). In particular, the number of DEGs in inbred parental line C seed was significantly lower than corresponding numbers found in other inbred parental lines. Notably, differences in transcript abundance between inbred parental lines for a given hybrid could be characterized as one of three difference types: expression abundance of the parental allele from one inbred line was greater than (>) that of the other, was less than (<) that of the other or was equal (=) to that of the other (Figure 1). In the present study, numbers of detected DEGs based on higher or lower mRNA-level abundance in leaf and ear organs of pairs of inbred parental maize lines were relatively similar. However, for some pairs of inbred lines as Z and C, C and M, C and Q, special ratios of parental gene expression levels in hybrid seeds relative to other tissues were observed as follows: N_*Z*__>__*C*_:N_*Z*__<__*C*_ ≈ 3:1, N_*C*__>__*M*_:N_*C*__<__*M*_ ≈ 1:3, N_*C*__>__*Q*_:N_*C*__<__*Q*_ ≈ 1:3. Thus, differences in allelic expression abundance between superior inbred parental lines and other lines suggests that superior inbred parental lines provide a more diverse and extensive selection background that supports a greater range of variation of gene expression in hybrid offspring. We might also infer from these results that hybrids with dominant gene action for a given trait are only produced when transcript abundance of genes derived from the superior inbred parental line reaches a certain proportion of the total transcript abundance derived from genes of both parental lines.

### Partial Parental Expression of Alleles in Six Hybrids

**FIGURE 2:**
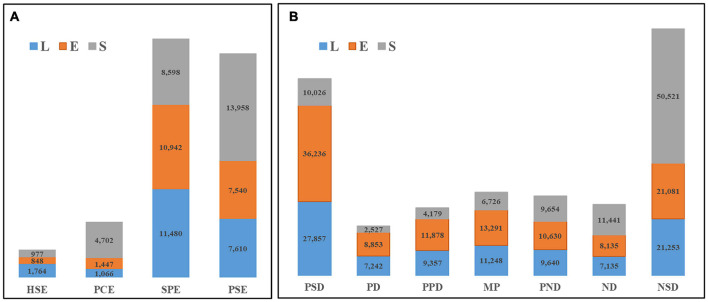
Gene expression pattern in hybrids. Qualitative changes (PAVs) and quantitative changes (PHCE) in gene differential expression were represented in panels **(A,B)**, respectively. The numbers in the bars represent the DEGs in different gene expression types (horizontal classification), and the different colors indicate that each tissue part is listed in the figure.

Notably, no significant differences were found among total numbers of genes expressed among different hybrids (Supplementary Figure 1). However, presence-absence variations (PAVs) of DEGs that were detected in the four parental lines appeared as qualitative differences in hybrid differential gene expression profiles, with PAVs accounting for 19.18% of all differences detected in hybrid transcript expression patterns. Of all PAVs, single-parent expression (SPE) and parent-specific expression (PSE) variations, the most extreme types of partial expression patterns involving high- or low-value parent alleles, were detected in DEGs in greatest proportions of 43.73 and 41.07%, respectively (Figure 2A). Expression patterns of most DEGs (80.82%) were of the parent-hybrid co-expression (PHCE) type (Supplementary Table 2). Among PHCE-type DEGs, we observed two predominant transgressive expression patterns, negative super-dominance (NSD) and positive super-dominance (PSD), with NSD and PSD proportions of all hybrid DEGs found to be 31.06 and 24.30%, respectively (Figure 2B). Therefore, we conclude that partial parental expression of alleles, which allele expressed biased toward to one of their parents, was responsible for qualitative variation in gene expression, while PHCE-type variations led to quantitative variation in gene expression. We thus presume that PHCE-type variations could be the main driving factor of heterosis expression in maize.

Partial parental expression of parental alleles in tissues of hybrids was consistent, but strength of partial expression was tissue-specific. With regard to qualitative variation, the number of SPE-DEGs with high parental partial expression exceeded the number of PSE-DEGs with low parental partial expression by 6.57%. Specifically, numbers of SPE-DEGs in leaves and ears were 50.85 and 45.12% greater, respectively, than corresponding numbers of PSE-DEGs, while in seeds the number of SPE-DEGs was only 61.60% of the number of PSE-DEGs. With regard to quantitative variation, the number of genes with expression biased toward that of the low-value parent (NSD-, ND-, and PND-DEGs) in hybrids was 26.52% higher than the number of genes whose expression was biased toward that of the high-value parent (PSD-, PD-, and PPD-DEGs). Notably, hybrids with biased gene expression toward alleles of the low-value parent exhibited significantly superior levels of quantitative variation.

Ultimately, both qualitative and quantitative variations were found to be associated with specific gene expression patterns in seeds as compared to leaves and ears, such as high proportions of PCE-, PSE- and NSD-DEGs and lower proportions of SPE-, PSD-, PD-, and PPD-DEGs in seeds as compared to corresponding proportions found in leaves and ears. Among qualitative variants, expression of alleles in hybrid seeds tended to be biased toward expression of low-value parent (PSE) alleles or was completely silent (PCE). However, with regard to quantitative variation, the number of NSD-DEGs in seeds was about 2.5 times the average number detected in leaves and ears, while the combined number of PSD-, PD- and PPD-DEGs in seeds was only 32.99% of the average number of DEGs in leaves and ears. Meanwhile, the number of DEGs exhibiting mid-parent allelic expression patterns was only about half of DEG numbers for other stages. Ultimately, hybrid gene expression patterns associated with the early reproductive growth stage significantly differed from patterns associated with other stages and were more highly influenced by alleles with partial to low parental expression patterns than by alleles with high parental expression.

### Specific Patterns of Gene Expression in the Zhengdan958

**FIGURE 3:**
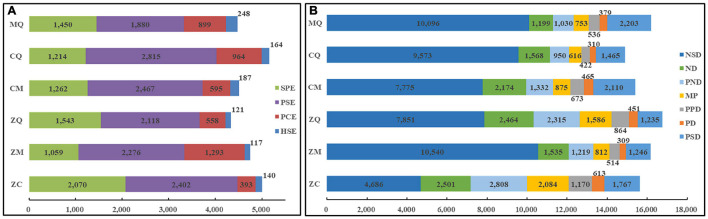
Gene expression patterns in seeds of hybrids. The differential expression of DEGs in seeds of hybrids with PAV **(A)** and PHCE patterns **(B)**, where the expression quantity of different types of genes is marked on the bar, and different colors in the figure represent different types of DEGs listed on the right side of the figure. The abscissa is the number of differentially expressed genes.

To further clarify genes associated with various expression patterns in hybrids, we compared expression profiles of various genes exhibiting PAV and PHCE expression patterns in different tissues of ZC and other hybrids. ZC seeds yielded the highest number of SPE-DEGs and lowest number of PCE-DEGs, in opposition to gene expression patterns of hybrids overall. Although DEGs in ZC seeds with PAV-type expression patterns did not have a significant quantitative advantage, the total number of SPE-DEGs was highest, comprising 41.36% of all PAVs, a proportion that was 58.55% higher than the average proportion of SPE-DEGs for all other hybrids (28.36%). However, the proportion of ZC seed PCE-DEGs was only 45.60% of the overall average proportion for other hybrids (Figure 3A), while ZC seed PHCE-type expression trends were opposite to overall gene expression trends in hybrids. Specifically, lowest numbers of NSD-DEGs were found in ZC seeds, which comprised only 51.12% of the average number of NSD-DEGs detected in all other hybrids. Meanwhile, ND-DEGs, PND-DEGs, MP-DEGs, PPD-DEGs and PD-DEGs numbers in ZC seeds significantly exceeded average numbers in other hybrid seeds by 39.88, 105.08, 124.47, 94.42, and 60.14%, respectively, (Figure 3B). No other notable associations were found in leaves and ears in this study except for a result indicating the number of PSD-DEGs in ears of ZC was only 2/3 that of ears of other hybrids (Supplementary Figure 2). Therefore, we conclude that unique distribution patterns of DEGs in ZC are highly relevant to ZC grain yield-related heterosis.

### Zhengdan 958-Specific Genes and Their Enrichment Pathway

**FIGURE 4:**
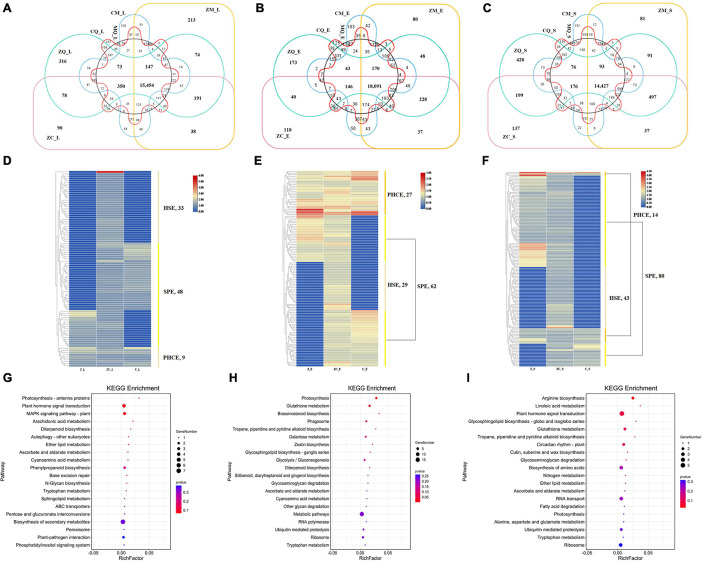
Gene expression specificity of Zhengdan958 in different tissues. The DEGs of Zhengdan958-specific genes in leaves **(A)**, ears **(B)** and seeds **(C)**, and their expression patterns in leaves **(D)**, ears **(E)** and seeds **(F)** and the enrichment biological pathways in leaves **(G)**, ears **(H)** and seeds **(I)**. The Venn diagrams **(A–C)** show the distribution of DEGs of Zhengdan958-specific genes, and the overlapping regions represent the genes that are co-expressed among/between hybrids. The transition of colors from red to blue in heat maps **(D–F)** shows that the expression abundance of DEGs changes from high to low, the color bands on the right indicate different gene expression types, and the number of genes is listed near the type names in the figures. The size of dots in the bubble charts **(G–I)** represents the number of genes, and the color of the dots representing the *P*-value which listed in the figures.

To determine factors unique to ZC as the dominant hybrid, we identified 90, 118 and 137 ZC-specific DEGs in leaves (Figure 4A), ears (Figure 4B) and seeds (Figure 4C), respectively, that were not co-expressed in different tissues (Supplementary Figure 3). Details of ZC-specific genes are listed in Supplementary Table 3. The results indicated that these ZC-specific genes were expressed exclusively in different tissues, with synergistic expression of these genes forming the molecular basis for ZC comprehensive superiority relative to other hybrids.

**FIGURE 5:**
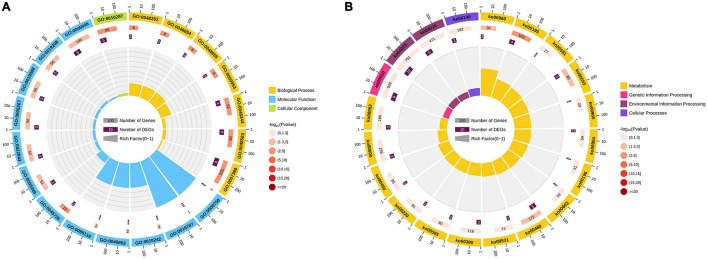
Enrichment circle diagram of Zhengdan958-specific genes. The Top-20 pathway circles of significant enrichment difference were drawn on all the Zhengdan958-specific genes based on GO **(A)** and KEGG **(B)** enrichment pathways. Four circles in the circle diagram from outside to inside. The first circle is the enriched classification, and outside the circle is the scale of the number of genes, different colors represent different classifications. The second circle is the number of background genes in this category, the more genes the longer the bar, the smaller the value the redder the color. The third circle shows the total number of target genes. Rich factor value of each classification in the fourth circle.

In leaves, 33 HSE-DEGs, 48 SPE-DEGs and 9 PHCE-DEGs were identified, including 4, 1, 1, 2 and 1 DEGs with NSD-, ND-, PND-, MP- and PSD-type expression patterns, respectively. Among these DEGs, one HSE-DEG, Zm00001d024372, showed significant expression activity (Figure 4D) and was found to encode chloroplastic chlorophyll a-b binding protein. Thus, we inferred that Zm00001d024372 was an important ZC leaf protogene. Results of subsequent functional enrichment analyses indicated that specific ZC leaf DEGs were mainly enriched in biological pathways plant hormone signal transduction, MAPK signaling pathway-plant and photosynthesis—antenna proteins (Figure 4G). In ears, 29 HSE-DEGs, 62 SPE-DEGs and 27 PHCE-DEGs, including 9, 6, 2, 3, 1 and 6 DEGs with NSD-, ND-, PND-, PPD-, PD- and PSD-type expression patterns (Figure 4E), were enriched for photosynthesis, glutathione metabolism and brassinosteroid biosynthesis pathways (Figure 4H). In seeds, 43 HSE-DEGs, 80 SPE-DEGs and 14 PHCE-DEGs, including 7, 4, 1 and 2 DEGs with NSD-, ND-, MP- and PSD-type expression, were identified (Figure 4F). These DEGs were mainly enriched for pathways plant hormone signal transduction, arginine biosynthesis, glutathione metabolism and other biological pathways (Figure 4I). Taken together, these results indicate that ZC-specific DEGs were significantly and generally enriched for multiple biological pathways that included photosynthesis, plant hormone signal transduction, biology metabolism of glutathione, tryptophan, and others and biosynthesis of tropane, piperidine, pyridine alkaloid and diterpenoid (Figure 5B).

Gene Ontology (GO) pathway analysis showed that functions of these genes were mainly related to two of the three main GO categories (Supplementary Table 4). In the category of biological process, genes detected within ZM00001D000361 (myb115), Zm00001d019230 (sid1), Zm00001d051465 (zag5), and ZM00001D053895 (bhlh51) play important roles in the morphogenesis of floral organs, such as the anther and anther wall tapetum. In the category of molecular function, the protein product of ZM00001D028264 participates in lipid IVA biosynthesis due to its UDP-3-*O*-[3-hydroxymyristoyl] *N*-acetylglucosamine deacetylase activity, the protein product of ZM00001D039634 (d1) participates in gibberellin biosynthesis due to its gibberellin 3-beta-dioxygenase activity, the protein product of Zm00001d036535 (oec33) has oxygen evolving activity, the protein product of Zm00001d048593 (rca1) has ribulose-1,5-bisphosphate carboxylase/oxygenase activator activity and the protein product of ZM00001D042122 participates in quercetin sulfate biosynthesis due to its brassinosteroid sulfotransferase activity (Figure 5A). Taken together, all of these protogene-associated enrichment pathways contribute to the overall dominance of ZC.

Among expression patterns of DEGs specifically expressed in each hybrid within the subset of PAV-type DEGs, SPE-DEGs showed a quantitative expression advantage over HSE-DEGs, while no notable variant-related expression advantage was found for PHCE-type DEGs (Supplementary Figure 4). These results thus suggest that impacts of advantageous effects due to dominant expression of PAV-type DEGs exceeded effects of co-dominant expression by hybrid-specific DEGs.

## Discussion

### Comparisons of Transcript Abundance Among Inbred Lines Reveal Parental Allele-Associated Complementation Effects

Differential gene expressions are thought to play important roles in plant phenotypic development (Wallace et al., 2014), with intergenerational differential gene expression producing hybrid phenotypes that are either superior or inferior to parental phenotypes (Stupar et al., 2008). Furthermore, enrichment in hybrids of advantageous DEGs associated with certain parental alleles may explain superior hybrid qualities (Shao et al., 2019). Given the strong correlation between differential gene expression and hybrid performance, hybrid viability can be predicted by examining transcriptional activity at parental level (Thiemann et al., 2010). Intriguingly, here the number of DEGs varied significantly in different tissues, with greater numbers of genes found to be expressed in young ears in both hybrids and inbred parental lines, while numbers of expressed genes were similar in leaves and grains (Supplementary Figure 1). This result suggests that differences in gene expression abundance of parental inbred lines benefits the hybrid when the total numbers of DEGs are consistent or nearly consistent among parents and hybrid offspring. Notably, here we discovered that differences in transcript abundance between parental inbred lines and hybrid offspring varied among hybrids. Specifically, in seeds of dominant hybrids, the proportion of expressed genes was skewed to represent greater expression abundance of transcripts from one parent (Figure 1). This “asymmetry” of gene expression abundance between parental inbred lines may support a wider range of gene expression complementarity in hybrid offspring that can appear as heterosis. Therefore, we support the idea that specific expression of different alleles leads to a broader plasticity of gene expression (Shao et al., 2019).

### Tissue-Specific Expression of Alleles Provides Opportunities for Heterosis at Various Stages

Tissue-specific genes refer to genes specifically expressed in different tissues that generate products conferring plants with specific morphological and structural characteristics and physiological functions. Tissue-specific gene expression can lead to phenotypic variation, especially in the case of dominant gene expression in hybrid tissues and organs that endows hybrids with special traits (Zhou et al., 2019). In this study, trends of variations of DEGs numbers among different tissues of hybrids and inbred parental lines were consistent (Supplementary Figure 1). Meanwhile, DEGs numbers varied significantly in different tissues, with numbers of genes expressed in ears significantly exceeding numbers expressed in leaves and seeds. This result could reasonably be attributed to the fact that switching of maize plants from vegetative growth to reproductive growth requires coordinated co-expression of a large number of genes.

In general, characterizations of tissue-specific expressed genes have improved our understanding of relationships between gene expression and tissue development (Xiao et al., 2010). Here, gene expression patterns differed significantly among different tissues. Regardless of whether the entire overall gene expression pattern of a hybrid resulted from qualitative variation or quantitative variation, significantly lower numbers of genes in hybrids exhibited expression patterns that were biased toward expression patterns of high-value parental genes than were biased toward expression patterns of low-value parental genes. We also found that hybrid seed-associated expression patterns significantly differed from expression patterns found in other tissues of hybrids, with numbers of PCE-DEGs, PSE-DEGs and NSD-DEGs detected in seeds that were respectively four times, two times and 2.5 times greater than corresponding numbers in ears or leaves. Meanwhile, numbers of PSD-DEGs, PD-DEGs, PPD-DEGs and MP-DEGs in seeds were respectively about one-third, one-third, one-half and one-half corresponding numbers associated with ears or leaves. Therefore, analysis of tissue-specific expression of DEGs in seeds, which represent the early reproductive growth stage, revealed underlying molecular mechanisms for heterosis in maize tissues that influenced grain yield, an observable trait. Validation of these results awaits further investigation.

### Gene Expression Patterns of Allele Confers Heterosis Intensity Changes

A plethora of studies based on comparisons of differential gene expression profiles between maize hybrids and their parental inbred lines have elucidated mechanisms responsible for maize hybrid vigor. In one study, about 8.9–15.3% of genes were differentially expressed between hybrid and parental inbred lines in maize embryos at 6 days post-pollination (Meyer et al., 2007). In another study, Stupar and Springer found that F_1_ progeny of inbred lines B73 and Mo17 exhibited significant differences in gene expression during seedling and young spike stages and in embryonic tissue as compared with expression patterns in parents (Stupar and Springer, 2006). In yet another study of the Zhongdan 808 hybrid, DEGs derived from parent NGS exhibited greater relative transcript abundance increases than did DEGs derived from parent CL11 (Hu et al., 2016), although these results were not related to the degree of heterosis, as studied in this work.

As mentioned above in the introduction section, we confirmed that the overall superior performance of ZC has led to large-scale ZC planting in China. To explore molecular mechanisms at the gene transcriptional level that are responsible for ZC superiority relative to other hybrids, we investigated ZC transcriptional gene expression patterns and compared them to expression patterns of other hybrids. Due to our main interest in grain yield-related heterosis, here we focused on ZC seed gene expression, not gene expression in leaves and ears, in order to study yield-related heterosis. Notably, ZC hybrid gene expression trends with regard to both qualitative and quantitative types of variation were diametrically opposed to trends observed for other hybrids, especially for expression trends in seeds (Figure 3 and Supplementary Figure 2).

Differential gene expressions exhibiting SPE- and PCE-type expression patterns comprised the highest and lowest proportions, respectively, of DEGs identified in ZC seeds and respectively exhibited greatest biases toward expression of high-value and low-value parental alleles. Of PAV-type DEGs, SPE-DEGs comprised the highest proportion and PCE-DEGs comprised the lowest proportion of average hybrid DEGs. Such gene expression trends may provide clues to understanding why PAV-type DEGs are predominant in hybrid ZC. Importantly, NSD-type variation was the least prevalent type of variation observed for quantitative variants, while all other expression pattern types, except for those with an overdominance expression pattern, showed obvious quantitative advantages in ZC seeds. Ultimately, preferential expression in ZC seeds of high-value parental genes exhibiting qualitative variation played an important role in dominant expression of the hybrid. Meanwhile, quantitative-type expression of genes of the ultra-low-value parent (such as NSD) were lowest, thus illustrating that preferential expression of superior alleles derived from high-value parents played an important role development of hybrid advantage, which was consistent with the previous research in rice (Huang et al., 2015). Thus, overall gene expression patterns in hybrids in combination with comparative advantages should be helpful for guiding future breeding programs.

### Enrichment of Superior Specific Alleles Promotes Heterosis

Differential gene expressions are thought to play important roles during plant phenotypic development (Wallace et al., 2014). Furthermore, connections among advantageous alleles of parental genes have been proposed to explain hybrid vigor (Shao et al., 2019). In other words, higher levels of parental gene expression in hybrids could be due to enrichment of advantageous DEGs. The most direct strategy for identifying differences at the molecular level between ZC and other hybrids would be to screen for ZC-specific DEGs within the set of DEGs of all hybrids. Here, we analyzed expression patterns and enrichment pathways associated with ZC-specific DEGs detected in different tissues (Figure 4). The number of DEGs with expression patterns aligning with the PAVs model exceeded the number aligning with the PHCE model, regardless of whether the DEGs were unique to ZC or other hybrids. While analyzing expression pattern of ZC-specific genes, we assumed that these genes were already expressed in ZC and therefore were not HSE-type and PCE-type genes. Obviously, DEGs with PAV type expression patterns comprised the largest proportion of genes with altered expression associated specifically with hybrids, including the ZC.

It is noteworthy that a tissue-specific gene expressed in leaves (Stelpflug et al., 2016; Hoopes et al., 2019), chloroplastic chlorophyll a-b binding protein-encoding gene (ZM00001D024372, Lhca1), was identified as having significantly higher activity in ZC tissues (Figure 4D). This finding was important in that light-harvesting protein complexes (LHC I) form proteins encoded by Lhca1 and other genes (Lhca2, Lhca3, Lhca4, etc.) to trap sunlight within photosystem I (PSI), a key photosynthetic system in higher plants (Qin et al., 2015). Our suggestion is that Lhca1 should be targeted as a potentially useful gene source as part of future maize molecular breeding strategies to boost grain yield of maize.

In biological systems, various genes perform biological functions in coordination with one another. Coordinated gene functions associated with DEGs can be elucidated using KEGG pathway analysis to identify pathways with significantly enriched DEGs representation as compared to the genomic background. Here we found that enriched pathways associated with ZC-specific DEGs varied among different tissues, including plant hormone signal transduction in leaves, glutathione metabolism in ears, and arginine biosynthesis in seeds, all of which were key nodes of interconnected ZC-specific key biological pathways. Obviously, the enrichment of protogenes gradually shifted from photosynthetic signal to regulation of plant hormones and then to biosynthesis of amino acids at different developmental stages of maize from vegetative growth center to reproductive growth center. Overall, enrichment of ZC-specific DEGs was associated with pathways related to photosynthesis, glutathione metabolism and plant hormone signal transduction contributed to comprehensive phenotypic expression associated with heterosis. These results were validated by GO enrichment analysis, in which numerous genes were found to be functionally associated with biological processes related to biological regulation and response to stimulus and to molecular functions associated with binding and catalytic activity. Thus, superior alleles accumulate in the process of biosynthesis improvement, thereby promotes heterosis.

## Conclusion

Heterosis has greatly boosted maize production and benefited human populations by providing greater food and economic security. Nevertheless, hybrid heterosis effects for a given combination of inbred parental lines cannot yet be predicted. In this study, six hybrids generated *via* incomplete diallel crosses of inbred parental lines were subjected to differential expression analysis to assess heterosis intensity. In general, the difference in gene expression abundance of alleles from parental inbred lines forms the basis of dominant performance of hybrids. Here, global gene expression patterns differed significantly between ZC and other hybrids, due to differences in both qualitative variation and quantitative variation of DEGs. By comparing gene expression patterns between ZC and other hybrids, the gene expression pattern of this strong predominant hybrid was revealed as was the important role of partial parental expression in heterosis. Meanwhile, we identified ZC-specifically expressed genes, as well as enrichment pathways associated with the set of identified genes. Annotation results indicated that enriched protogenes were associated with multiple biological pathways, such as photosynthesis, plant hormone signal transduction, ion channel regulation and other pathways associated with binding and catalytic activities. Intriguingly, we found that synergistic effects of expression of multiple distinct tissue-specific genes in ZC promoted comprehensive heterosis. This study provides new perspectives for elucidating molecular mechanisms that underlie heterosis and can serve as a technical reference for designing breeding programs that harness strong heterosis effects to improve maize and other crops.

## Materials and Methods

### Plant Materials and Growth Conditions

Four elite maize inbred lines, Zheng58, Chang7-2, Mo17, and Qi319, were the typical lines of Reid, Sipingtou, Lancaster and P group, respectively. Six their hybrids, Zheng58 × Chang7-2, Zheng58 × Mo17, Zheng58 × Qi319, Chang7-2 × Mo17, Chang7-2 × Qi319, and Mo17 × Qi319, based on an incomplete diallel cross (NCII) were planted in April of 2019 at the Changping Station at the Institute of Crop Science, Chinese Academy of Agricultural Sciences (116°13′ E, 40°15′ N) in Beijing, China. Among these hybrids, Zhengdan958 is a commercially successful hybrid variety from Chinese maize breeding programs with the strong heterosis of yield (Li et al., 2012; Kogelman et al., 2014).

Tissue samples were collected as follows: (1) for leaf samples, three top leaves were collected from five-leaf stage seedlings and pooled; (2) for ear samples, more than 10 young ear tissues were collected at the stage of spikelet differentiation based on the phenotypic identification under a microscope and pooled; and (3) for seed samples, developing kernels containing embryo in the middle of ear were collected at 15 days after pollination (DAP). For each tissue, we sampled six biological replicates, three of which were used for subsequent analysis while the rest served as a backup. Tissues were sampled, frozen immediately in liquid N_2_, and then stored at −80°C until being used for RNA extraction.

For the convenience of subsequent description, the names of each maize line, hybrid, and sampled tissue are abbreviated as follows: Zheng58 (Z), Chang7-2 (C), Mo17 (M), Qi319 (Q), Zheng58 × Chang7-2 (ZC), Zheng58 × Mo17 (ZM), Zheng58 × Qi319 (ZQ), Chang7-2 × Mo17 (CM), Chang7-2 × Qi319 (CQ), Mo17 × Qi319 (MQ), Leaf (L), Ear (E), and Seed (S). Thus, the first biological replicate of a leaf sampled from the Zheng58 × Chang7-2 (ZC) hybrid is abbreviated as ZC_L1.

### Construction and Sequencing of mRNA Libraries for RNA-Seq Analysis

A total of 90 samples of maize tissue (10 genotypes × 3 tissues × 3 biological replicates) were qualified for RNA sequencing (RNA-Seq) library construction. Tissues at the same stage of development were sampled from different plants and mixed to diminish differences between genotypes caused by sampling times.

We extracted total RNA with TRIzol Reagent (Invitrogen, CA, United States) according to the manufacturer’s recommendations. The quantity and purity of total RNA samples were analyzed on the Bioanalyzer 2100 and RNA 1000 Nano Lab Chip Kit (Agilent, CA, United States) with RIN number > 7.0. We purified poly(A +) RNA from 5 mg of total RNA by two purification rounds on poly-T oligo magnetic beads. After mRNA was purified, it was fragmented in a fragmentation buffer containing divalent cations at high temperature. We then reverse-transcribed the RNA fragments following the procedures in the mRNA-Seq sample preparation kit (Illumina, San Diego, CA, United States) to generate the cDNA libraries. The libraries were then subjected to paired-end sequencing (2 bp × 150 bp, PE150) on an Illumina HiSeq 4000 platform at LC-BIOTECHNOLOGIES (LC Sciences, United States) Co., Ltd.,^1^ according to the company’s recommendations.

### Analysis of Illumina RNA-Seq Data

We sequenced the transcriptomes of each genotype and tissue combination and generated a total of 3,899 billon raw paired-end 150-bp Illumina reads comprising a total of 584.86 gigabases (Gb) of sequence. We aligned the raw reads from each sample to the B73 maize genome^2^ using Hisat2 (version 2.0.5) (Kim et al., 2016), which excludes some reads based on quality data and maps high-quality reads to the reference genome, with a minimum intron length of 20 bp and a maximum intron length of 50 kb, and other parameters set to defaults. We removed low-quality reads that contained sequencing adaptors or sequencing primers, reads containing more than 5% unascertained bases, and bases below Q20 before assembly. Up to 20 multiple alignments and at most two mismatches per read were allowed in HISAT when we mapped reads to the B73 reference genome. We used StringTie (version 1.3.4d) (Pertea et al., 2015) to calculate FPKM values (fragments per kilobase of exon model per million mapped reads) for RNAs in each sample. The genes with FPKM less than 1 were defined as non-expressed genes, so they were deleted in the calculation process. We used the R Bioconductor package “Mfuzz” (Kumar and Futschik, 2007) to cluster expressed genes according to the expression profiles of the parent-hybrid triplets. Short-read transcripts were reconstructed using Cufflinks (version 2.0) (Trapnell et al., 2012) and Trinity (version 0.1.0-alpha.14) (Haas et al., 2013), and then were mapped to the B73 maize genome using GMAP (Thomas and Serban, 2010) and were filtered for alignment coverage > 85% and alignment identity > 90%. A total of 3,257 billon cleaned paired-end reads comprising 488.57 Gbp of valid sequence data were produced. The results of sequence statistics and quality control were list in Supplementary Table 1. We have submitted the raw sequence data to NCBI (BioProject: PRJNA682889), and which has yet to be released.

### Functional Enrichment Analysis

Enrichment analyses of DEGs were conducted using FuncAssociate 3.0 (Berriz et al., 2009) using Ensembl gene identifiers. GO enrichment analysis was performed using the OmicShare tools, a free online platform for data analysis^3^. Firstly, all DEGs were mapped to GO terms in the Gene Ontology database^4^, gene numbers were calculated for every term, significantly enriched GO terms in DEGs comparing to the genome background were defined by hypergeometric test. The calculated *p*-value was gone through FDR correction, taking FDR ≤ 0.05 as a threshold. GO terms meeting this condition were defined as significantly enriched GO terms in DEGs. We used the ggplot2 program package to display the results of GO (Gene ontology) and KEGG (Kyoto Encyclopedia of Genes and Genomes,^5^) enrichment analyses. In addition, KO (KEGG Orthology) labels were used to represent the classification of proteins with similar functions in the same pathway.

### Identification of Gene Expression Patterns

For each triplet comprising the set of parents and their hybrid, differences in gene expression could follow one of five patterns: (1) parental co-silence expression (PCE), in which the genes are expressed in parents but not in their hybrid; (2) parent-specific expression (PSE), in which genes are expressed in only one parent but not in the other parent and their hybrid; (3) hybrid-specific expression (HSE), in which genes are expressed only in the hybrid but not in its parents; (4) single-parent expression (SPE), in which genes are expressed in hybrid and one of its parents; and (5) parental-hybrid co-expression (PHCE), in which genes are expressed in the hybrid and both of its parents.

Models 1–4 (PCE, PSE, HSE and SPE) represent qualitative variations in differential gene expression that are essentially similar to presence-absence variations (PAVs) except that the gene is present in each genotype but are expressed only in a certain individual or genotype and not in another. As in the quantitative gene expression pattern (PHCE) model, hybrids can be divided into three categories (high-value parents, middle-value parents, and low-value parents) and seven subcategories according to their A value (Stupar and Springer, 2006), where:


$$A=(P_{\max}-F_{1})/\left(P_{\max}-P_{\min}\right)$$

In the formula, P_*max*_, P_*min*_, and F_1_ represent levels of the parent with higher gene expression, the parent with the lower expression, and that of their hybrid, respectively. The categories of high-value parents, middle-value parents, and low-value parents include 2 (A < 0.0, 0 ≤ A < 0.2), 3 (0.2 ≤ A < 0.4, 0.4 ≤ A < 0.6, 0.6 ≤ A < 0.8), and 2 (0.8 ≤ A < 1.0, A ≥ 1.0) subcategories with the A values corresponding to the interval in parentheses (Trapnell et al., 2009). Then all the genes of the aforementioned PHCE gene sets expressed in hybrids were divided into seven groups corresponding to positive super-dominance (PSD), positive dominance (PD), partial positive dominance (PPD), mid-parent (MP), partial negative dominance (PND), negative dominance (ND), and negative super-dominance (NSD) gene action.

## Data Availability Statement

The datasets presented in this study can be found in online repositories. The names of the repository/repositories and accession number(s) can be found below: https://www.ncbi.nlm.nih.gov/genbank/, PRJNA682889.

## Author Contributions

XL, JWe, and JWu conceived and designed the experiments. ML, ZX, JWe, and JH conducted the field experiments and collected the tissue samples. JWu and QZ performed the experiments and analyzed the data. JWu interpreted the data and drafted the manuscript. XL, JWe, and ML supervised the project and revised the manuscript. ZH, HY, DS, ZZ, XZ, and DZ provided helpful discussions on the manuscript. All authors read and approved the final manuscript.

## Conflict of Interest

The authors declare that the research was conducted in the absence of any commercial or financial relationships that could be construed as a potential conflict of interest.

## Publisher’s Note

All claims expressed in this article are solely those of the authors and do not necessarily represent those of their affiliated organizations, or those of the publisher, the editors and the reviewers. Any product that may be evaluated in this article, or claim that may be made by its manufacturer, is not guaranteed or endorsed by the publisher.

## Footnotes

1
www.lc-bio.com/

2
ftp://ftp.ncbi.nlm.nih.gov/genomes/genbank/plant/Zea_mays/latest_assembly_versions/GCA_000005005.6_B73_RefGen_v4

3
www.omicshare.com/tools

4
http://www.geneontology.org/

5
www.genome.jp/kegg

## Supplementary Material

Supplementary Figure 1 Number of DEGs in hybrids and their parental inbred lines among tissues. Histogram depicting the numbers of DEGs in each genotype (inbred lines and hybrids) and tissues [leaves (L), ears (E), and seeds (S)] with colors listed on the right. The columns in the histogram are divided in two sections by wide gaps. The bottom half depicts the number of DEGs in L, E, and S of maize inbred lines Z, C, M, and Q, which represent Zheng58, Chang7-2, Mo17, and Qi319. The top half depicts the number of DEGs in L, E, and S of the hybrids, which include Zheng58 × Chang7-2 (ZC), Zheng58 × Mo17 (ZM), Zheng58 × Qi319 (ZQ), Chang7-2 × Mo17 (CM), Chang7-2 × Qi319 (CQ), and Mo17 × Qi319 (MQ). The different inbred and hybrid genotypes are shown on the *y*-axis, and the numbers of significant DEGS expressed by the different genotypes are shown on the *x*-axis.

Supplementary Figure 2 Gene expression patterns in leaves and ears of hybrids. The differential expression of DEGs in leaves of hybrids with PAV **(A)** and PHCE patterns **(B)**, and the PAV **(C)** and PHCE **(D)** patterns in ears, where the expression quantity of different type of genes is marked on the bar, and colors in the figure represent different types of DEGs listed on the right side of the figure.

Supplementary Figure 3 Distributions of ZC-specific DEGs in tissues. Venn diagram with colors represent leaves (L), ears (E), and seeds (S), the numbers of DEGs are listed in the corresponding section.

Supplementary Figure 4 Expression patterns of specific genes expressed in different hybrids. The differential expression of DEGs in leaves (L), ears (E), and seeds (S) of hybrids with PAV **(A)** and PHCE **(B)** patterns. The composition of genes with different expression patterns in PHCE in leaves (L), ears (E) and seeds (S) was calculated in proportion to the total genes, respectively. Six hybrids: Zheng58 × Chang7-2 (ZC), Zheng58 × Mo17 (ZM), Zheng58 × Qi319 (ZQ), Chang7-2 × Mo17 (CM), Chang7-2 × Qi319 (CQ), and Mo17 × Qi319 (MQ).

Supplementary Table 1 Descriptive statistics and quality control information for RNA-Seq data. Genotypes and tissue are abbreviated as follows: Zheng58 (Z), Chang7-2 (C), Mo17 (M), Qi319 (Q), Zheng58 × Chang7-2 (ZC), Zheng58 × Mo17 (ZM), Zheng58 × Qi319 (ZQ), Chang7-2 × Mo17 (CM), Chang7-2 × Qi319 (CQ), Mo17 × Qi319 (MQ), Leaf (L), Ear (E) and Seed (S). Thus, the first biological replicate of a leaf sampled from the Zheng58 × Chang7-2 (ZC) hybrid is abbreviated as ZC_L1. All genotypes were compared to the B73 maize genome with the mapped reads and ratio in the last column.

Supplementary Table 2 Gene expression patterns in hybrids. Gene expressed with qualitative (PAVs) and quantitative (PHCE) variation in different tissues [leaves (L), ears (E), and seeds (S)] in the hybrids, which include Zheng58 × Chang7-2 (ZC), Zheng58 × Mo17 (ZM), Zheng58 × Qi319 (ZQ), Chang7-2 × Mo17 (CM), Chang7-2 × Qi319 (CQ), and Mo17 × Qi319 (MQ). The Gene expression patterns listed include different gene actions of parental co-silence expression (PCE), parent-specific expression (PSE), hybrid-specific expression (HSE), single-parent expression (SPE), negative super-dominant (NSD), negative dominant (ND), partial negative dominant (PND), mid-parent (MP), partial positive dominant (PPD), positive dominance (PD), and positive super-dominance (PSD).

Supplementary Table 3 Details of Zhengdan958-specific DEGs. Distribution, expression patterns and enrichment pathways of Zhengdan958-specific genes in different tissues (leaves, ears, and seeds). The Gene expression patterns (Models) listed include different gene actions of hybrid-specific expression (HSE), single-parent expression (SPE), and parental/hybrid co-expression (PHCE).

Supplementary Table 4 Details of Zhengdan958-specific genes in Gene ontology. Gene Ontology analysis were conducted with three categories of Biological Process, Cellular Component and Molecular Function in GO Term (level 1) by the Zhengdan958-specific genes, which were describes the molecular functions the gene products may perform, the cellular environment in which they are exposed, and the biological processes involved. The genes were assigned to the GO Term (level 2) listed with the rank in order of most to least of gene numbers.

## Abbreviations

QTLs: quantitative trait loci
DEGs: differentially expressed genes
PCE: parental co-silence expression
PSE: parent-specific expression
HSE: hybrid-specific expression
SPE: single-parent expression
PAVs: presence-absence variations
PHCE: parent-hybrid co-expression
PSE: parent-specific expression
NSD: negative super-dominance
ND: negative dominance
PND: partial negative dominance
MP: mid-parent
PPD: partial positive dominance
PD: positive dominance
PSD: positive super-dominance.
