# Supplementary figures and images for: Transcriptome Reveals Allele Contribution to Heterosis in Maize
Source: Front Plant Sci. 2021 Sep 23;12:739072. doi: 10.3389/fpls.2021.739072 (PMC8494984; doi:10.3389/fpls.2021.739072)

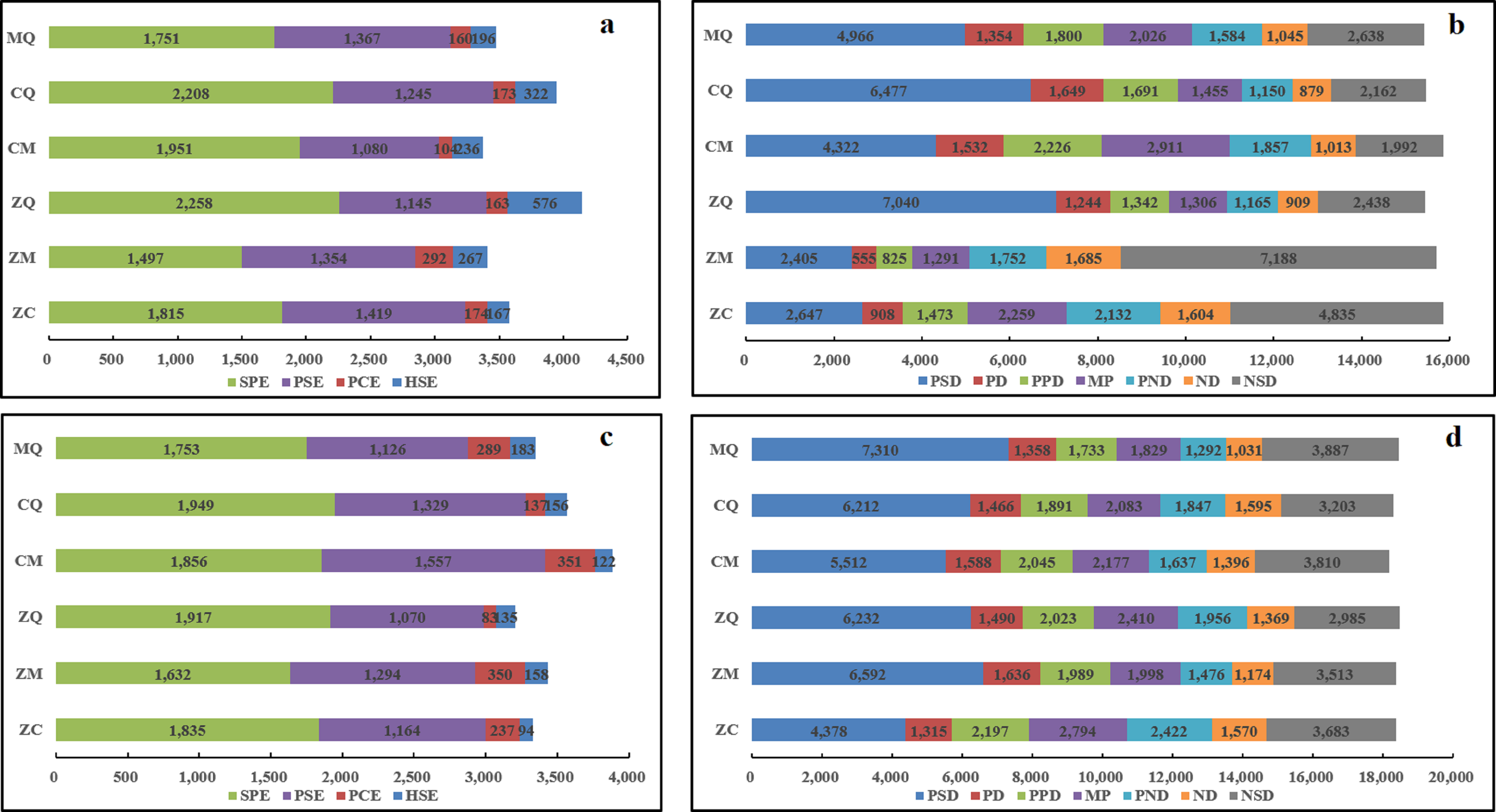

Supplement: Supplementary Figure 2 — Gene expression patterns in leaves and ears of hybrids. The differential expression of DEGs in leaves of hybrids with PAV (A) and PHCE patterns (B), and the PAV (C) and PHCE (D) patterns in ears, where the expression quantity of different type of genes is marked on the bar, and colors in the figure represent different types of DEGs listed on the right side of the figure. [file Image_2.JPEG]

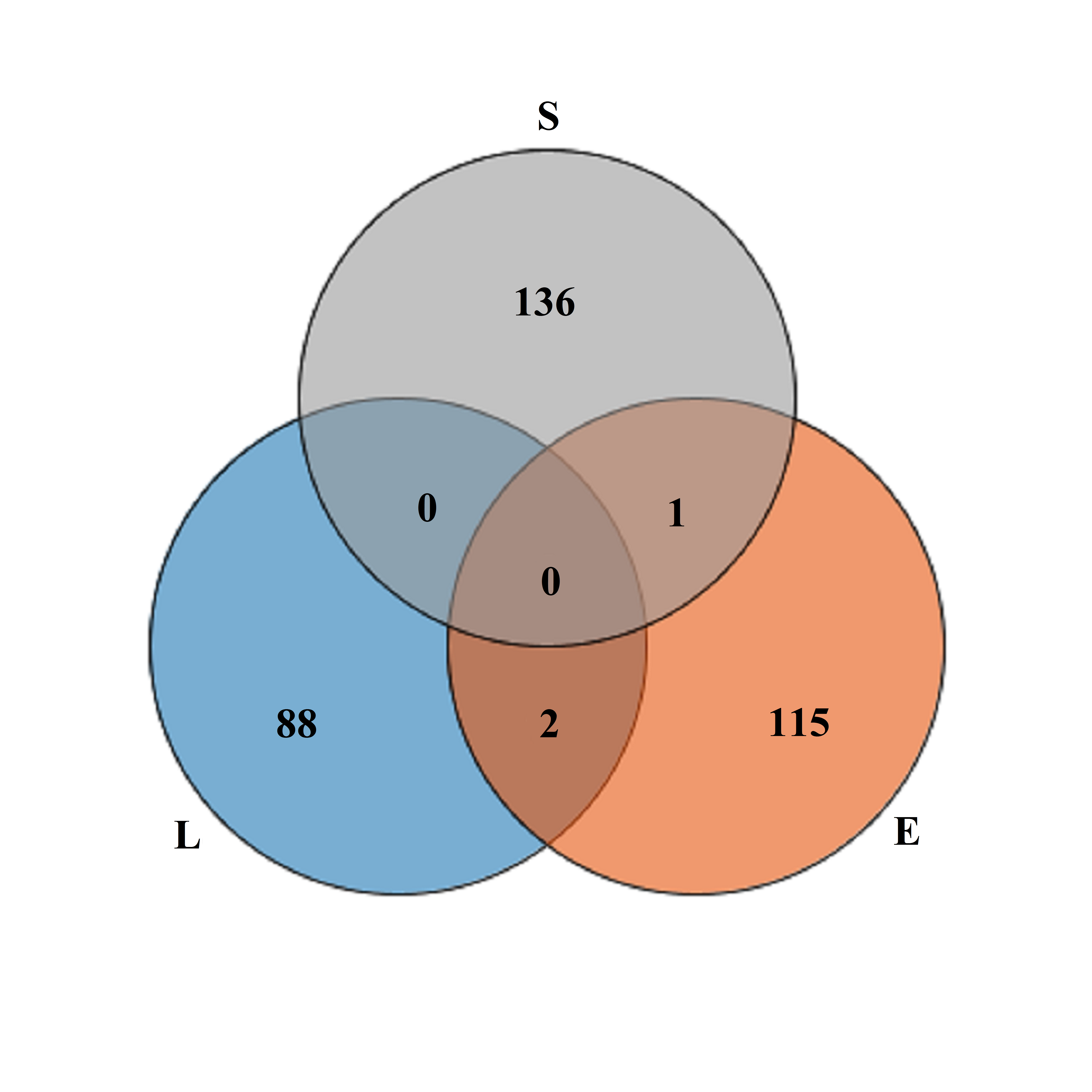

Supplement: Supplementary Figure 3 — Distributions of ZC-specific DEGs in tissues. Venn diagram with colors represent leaves (L), ears (E), and seeds (S), the numbers of DEGs are listed in the corresponding section. [file Image_3.JPEG]

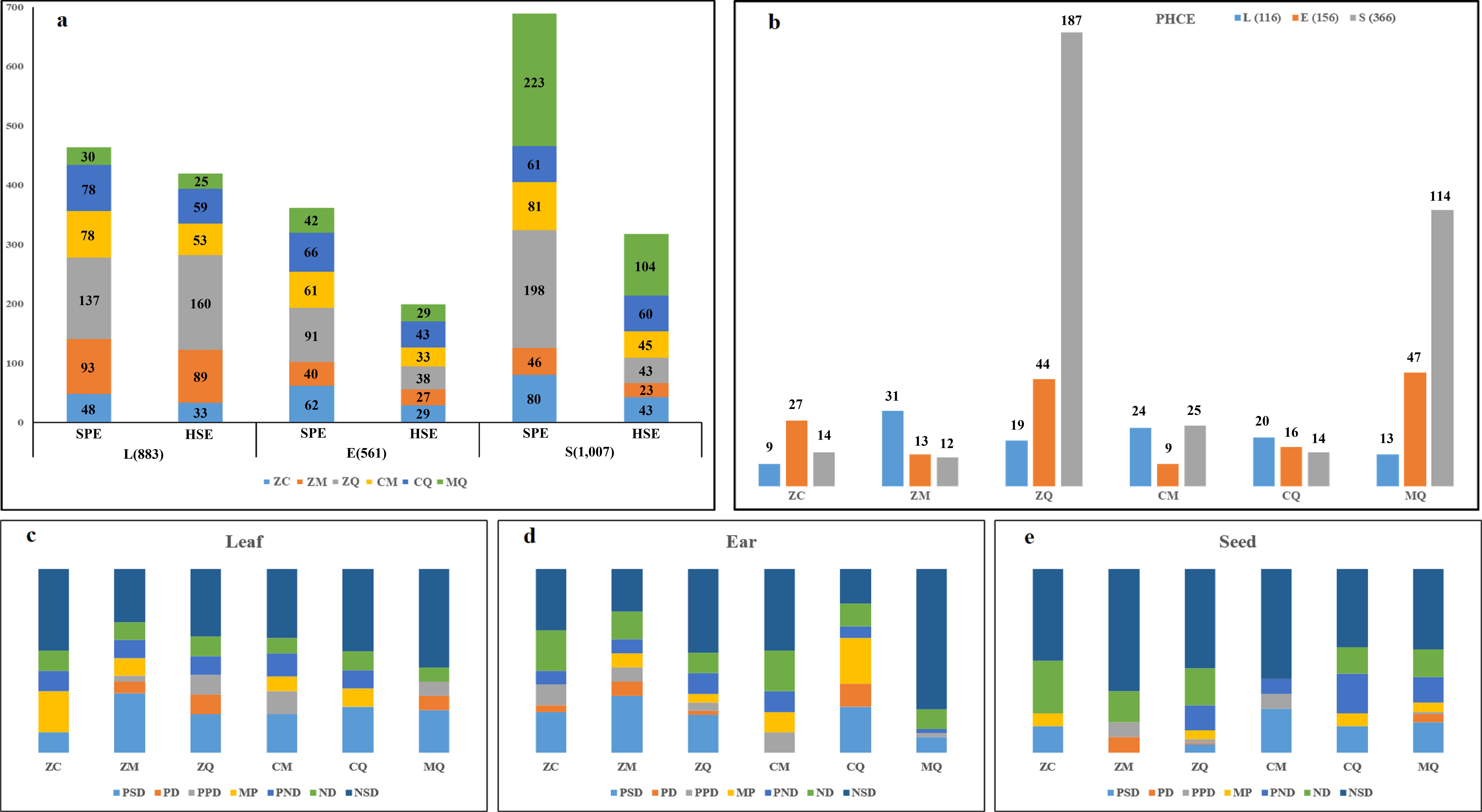

Supplement: Supplementary Figure 4 — Expression patterns of specific genes expressed in different hybrids. The differential expression of DEGs in leaves (L), ears (E), and seeds (S) of hybrids with PAV (A) and PHCE (B) patterns. The composition of genes with different expression patterns in PHCE in leaves (L), ears (E) and seeds (S) was calculated in proportion to the total genes, respectively. Six hybrids: Zheng58 × Chang7-2 (ZC), Zheng58 × Mo17 (ZM), Zheng58 × Qi319 (ZQ), Chang7-2 × Mo17 (CM), Chang7-2 × Qi319 (CQ), and Mo17 × Qi319 (MQ). [file Image_4.JPEG]
